# Oleic Acid Levels in HSA^LR^ Mouse Model of Myotonic Dystrophy Type 1

**DOI:** 10.3390/ijms27104211

**Published:** 2026-05-09

**Authors:** Dulce Peris-Moreno, Maria Sabater-Arcis, Nerea Moreno, Luis Orduña, Arturo López-Castel, Ariadna Bargiela, Ruben Artero

**Affiliations:** 1 CIBERER ISCIII, Avenida Monforte de Lemos 3-5, 28029 Madrid, Spain; dulce.peris@uv.es (D.P.-M.); arlocas@uv.es (A.L.-C.); ruben.artero@uv.es (R.A.); 2 Human Translational Genomics Group, University Institute for Biotechnology and Biomedicine (BIOTECMED), University of Valencia, Avenida Doctor Moliner 50, 46100 Valencia, Spain; 3 INCLIVA Biomedical Research Institute, Avenida Menendez Pelayo 4, 46010 Valencia, Spain

**Keywords:** oleic acid, myotonic dystrophy type 1 (DM1), HSA^LR^, *Sdc1*, transcriptomics, fatty acids

## Abstract

Myotonic dystrophy type 1 (DM1) is characterized by altered RNA processing, muscle wasting and metabolic dysregulation. In a previous in vitro study, we found reduced endogenous oleic acid (OA; 18:1n-9) in myogenic cells from DM1 patients, and short-term OA supplementation rescued key disease readouts. Here, we asked whether lipid pathways are perturbed in a DM1 in vivo background. We employed the widely used HSA^LR^ mouse model, which expresses human skeletal alpha-actin transcripts harbouring expanded CTG repeats and recapitulates key DM1 phenotypes. RNA-seq-derived functional enrichment across three independent HSA^LR^ datasets revealed a consistent enrichment of downregulated GO terms related to fatty-acid metabolism, suggesting impaired lipid handling. Guided by transcriptomic evidence of fatty-acid pathway downregulation, we quantified endogenous OA in HSA^LR^ mice versus FVB controls by LC-MS across sex, age (postnatal day 21 and 4 months) and tissue (quadriceps, gastrocnemius, and plasma), reporting values per tissue weight and per total protein. OA showed selective, context-dependent reductions in skeletal muscle, varying with tissue, sex, age and normalization, with the clearest deficits in males and in the gastrocnemius, while no generalized deficit was observed. Plasma OA was transiently higher at P21 in both sexes, consistent with the oleate-rich weaning mice diet. Together, the RNA-seq enrichment signal for fatty-acid pathways and the context-dependent OA changes point to network-level control (substrate supply, partitioning/transport, cofactors or downstream utilization), positioning OA as a potential measurable candidate marker of DM1-like muscle pathology in HSA^LR^ mice.

## 1. Introduction

DM1 is the most common adult-onset muscular dystrophy, caused by a CTG trinucleotide repeat expansion in the 3′ untranslated region of the *DM1 protein kinase* (*DMPK*) gene [1,2]. This mutation leads to the nuclear accumulation of toxic RNA transcripts, which sequester MBNL proteins, resulting in widespread alternative splicing defects [3,4]. Although DM1 predominantly affects skeletal muscle, this multisystemic disease is also characterized by myotonia, cardiac conduction abnormalities, and metabolic disturbances, including insulin resistance and dyslipidemia [5].

Beyond the canonical splicing defects, metabolic dysfunction is increasingly recognized as a core component of DM1 pathology. Clinical and experimental studies have reported impairments in glucose tolerance/insulin signalling, mitochondrial oxidative metabolism, and lipid/fatty-acid homeostasis [6,7,8,9]. Among fatty-acids, oleic acid (OA), a monounsaturated omega-9 fatty-acid (MUFA), plays a critical role in membrane dynamics, signalling, inflammation, and energy homeostasis [10,11] and protects against oxidative stress and lipotoxicity in various cellular contexts [12,13]. Recent work in DM1 patient-derived muscle cell models demonstrated reduced endogenous OA and the benefits of short-term OA supplementation [14]. However, whether OA is altered in vivo across relevant DM1 tissues, ages, and disease stages remains unresolved, and this is pivotal because a disturbed MUFA balance could affect pathways governing proteostasis and myogenic quality control as seen in metabolic disease pathogenesis or muscle atrophy [13,15,16].

In line with this possibility, autophagy dysregulation has been implicated in muscle wasting in DM1 animal models and patient-derived cells [17] which have revealed that miR-7 functions as a node linking myogenesis and autophagy. Specifically, miR-7 is reduced in DM1 tissues and models, and its restoration rescues myogenic defects while restraining autophagy hyperactivation [18,19,20]. Mechanistically, the RNA-binding protein Musashi-2 (MSI2), together with HuR, binds the pri-miR-7 terminal loop to suppress miR-7 biogenesis [21]. Importantly, OA facilitates miR-7 processing by remodelling the HuR/MSI2–pri-miR-7 complex [22] and OA binds to the N-terminal RNA-recognition motif of Musashi proteins, supporting family-level lipid sensing [23]. Taken together, these observations suggest that an OA shortfall would exacerbate MSI2-mediated repression of miR-7, thereby sustaining autophagy hyperactivation and impairing myogenesis, and thus provide a mechanistic bridge from metabolic disturbance to post-transcriptional pathology in DM1.

Consistent with this framework, our recent in vitro study found that OA levels are reduced in DM1 patient-derived myoblasts and myotubes, and that supplementation with OA was sufficient to reverse multiple pathological features such as impaired myotube growth, reduced fusion index, and autophagy hyperactivation [14]. Moreover, we reported decreased expression of stearoyl-CoA desaturase 1 (SCD1), the rate-limiting enzyme in OA biosynthesis, providing a possible explanation for the observed OA deficiency [16,24].

Despite these promising in vitro results, the in vivo status of OA in DM1 skeletal muscle remains largely unexplored. To address this gap, we re-analyzed publicly available RNA-seq datasets to assess whether fatty-acid metabolism is affected in HSA^LR^ muscle and we quantified endogenous OA levels in the skeletal muscle and plasma of HSA^LR^ mice, a transgenic model expressing expanded CTG repeats in human skeletal alpha-actin transcripts that recapitulates hallmark features of DM1 [25,26], evaluating age- and sex-dependent differences.

## 2. Results

### 2.1. Transcriptomic Downregulation of Fatty-Acid Metabolic Pathways in HSA^LR^ Muscle

First, we examined the expression of genes involved in fatty-acid metabolism in the HSA^LR^ mouse model of DM1 to validate prior observations in patient cells [14]. We re-analyzed three public RNA-seq datasets of male HSA^LR^ vs. FVB: two differential-expression comparisons of the quadriceps from young, sex-matched males of mice < 4 months [27,28] and one gastrocnemius comparison from 10- to 12-week-old males [29].

**Figure 1:**
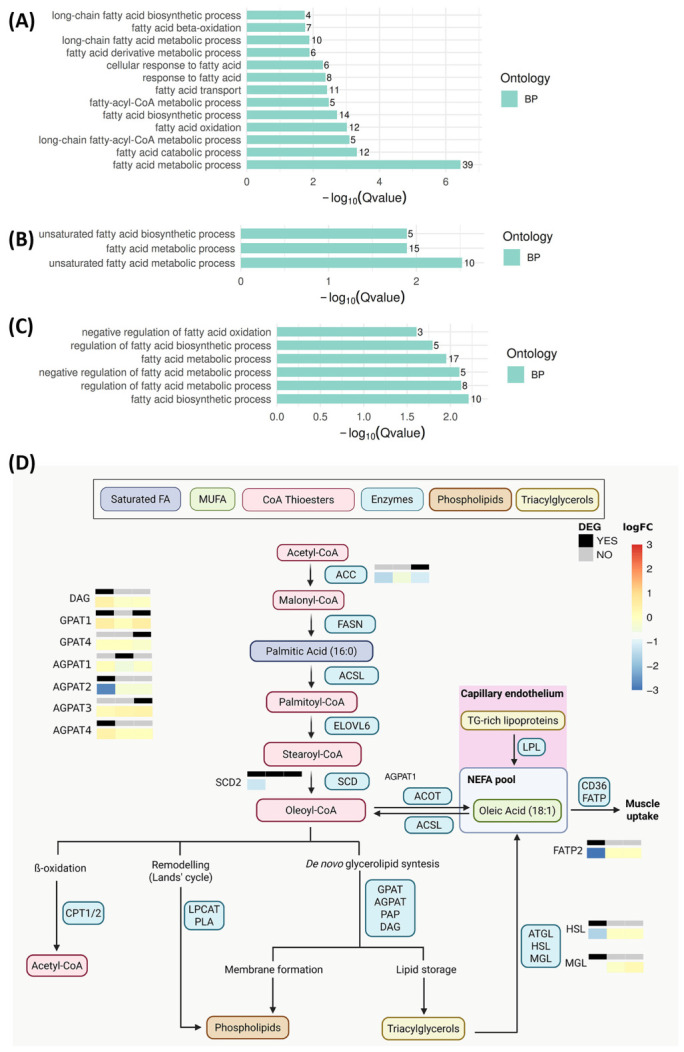
RNA-seq-derived functional enrichment of fatty-acid metabolic processes in HSA^LR^ muscle. (**A**–**C**) GO Biological Process (BP) enrichment of downregulated genes from HSA^LR^ vs. FVB differential-expression analyses. Dataset (**A**) [27] and (**B**) [28]: quadriceps, mice < 4 months, sex-matched. (**C**) Dataset [29]: gastrocnemius, mice 10–12-week-old males. Bars show −log_10_(q-value) (Benjamini–Hochberg FDR). Numbers on bars indicate gene counts per term. Only significant terms (FDR < 0.05) are shown. (**D**) Integration of differentially expressed genes (DEGs) into an oleic acid (18:1)/glycerolipid metabolism network schematic. Mini heatmaps adjacent to enzymes summarize log_2_ fold changes for each dataset comparison (left to right: datasets [27,28,29]). The colour denotes the direction and magnitude of log_2_FC, and the header indicates whether the gene was called differentially expressed in that comparison. Transcripts lacking mini heatmaps did not meet DEG criteria in any of the three analyses. The pathway scaffold (enzyme–metabolite relationships) follows curated pathway resources and the canonical fatty-acid metabolism literature [16,30,31,32], with RNA-seq expression changes overlaid from the re-analyzed datasets.

Functional enrichment of these comparisons (Figure 1A–C; FDR-adjusted q-values) showed that fatty-acid metabolism-related GO terms were enriched among downregulated genes, including the parent term “fatty-acid metabolic process” and multiple child processes such as β-oxidation, fatty-acid transport, (long-chain) fatty-acyl-CoA metabolism, fatty-acid biosynthesis, and unsaturated fatty-acid metabolism/biosynthesis. Regulatory categories such as “negative regulation of fatty-acid oxidation” and “regulation of fatty-acid biosynthetic process” were likewise enriched, reinforcing an overall transcriptional downshift in lipid metabolic capacity.

To identify drivers and interpret these changes mechanistically, we integrated the differentially expressed genes into a pathway view of OA and glycerolipid metabolism (Figure 1D) using curated pathway resources and canonical reviews that highlighted dysregulated nodes directly connected to MUFA and OA biosynthesis, consistent with constrained OA biosynthetic capacity. In this schematic, the mini heatmaps next to each enzyme summarize the three-dataset comparison [27,28,29], with colour indicating the direction and magnitude of the logFC and the black/grey header denoting whether the gene was declared as differentially expressed in that comparison. The MUFA-generating step (stearoyl-CoA → oleoyl-CoA) showed the most consistent signal, with reduced *Scd2* (negative log_2_FC across comparisons), whereas upstream *de novo* lipogenesis (*Acc* → malonyl-CoA) exhibited comparatively modest changes, suggesting limited remodelling at the level of palmitate synthesis. Within the glycerolipid synthesis arm (*Gpat*/*Agpat*/*Dag*-associated steps), most enzyme-encoding transcripts displayed small effect sizes with significance restricted to specific comparisons, although *Agpat2* showed a pronounced decrease in one comparison. Finally, genes involved in fatty-acid cellular uptake (via *Fatps*) and intracellular lipid mobilization (*Hsl*/*Mgl*) showed comparison-dependent decreases, consistent with altered availability and/or utilization of the non-esterified fatty-acid (NEFA) pool supplying muscle lipid metabolism across the three re-analyzed datasets.

These RNA-seq analyses demonstrate a transcript-level downregulation of fatty-acid metabolic pathways in HSA^LR^ skeletal muscle across three independent datasets (two quadriceps and one gastrocnemius; all male post-weaning mice). Beyond the parent term “fatty-acid metabolic process”, leading-edge genes consistently mapped to the Δ9-desaturation node, with *Scd2* showing reduced expression across datasets, whereas *Scd1* expression was comparatively unchanged. Together, these patterns localize the dominant transcriptional signal to the MUFA-generating step rather than to upstream *de novo* lipogenesis or global fatty-acid pathway repression.

### 2.2. OA Levels in HSA^LR^ DM1 Model Are Tissue-, Sex-, Age- and Normalization-Dependent

Following the RNA-seq analyses indicating reduced expression of genes annotated to fatty-acid metabolic processes in HSA^LR^ muscle, we quantified endogenous OA to test whether this component is systemically altered in vivo (as suggested by prior work in patient cells [14]) and to evaluate the effects of sex, age, tissue, and normalization strategy (per muscle weight versus total protein).

**Figure 2:**
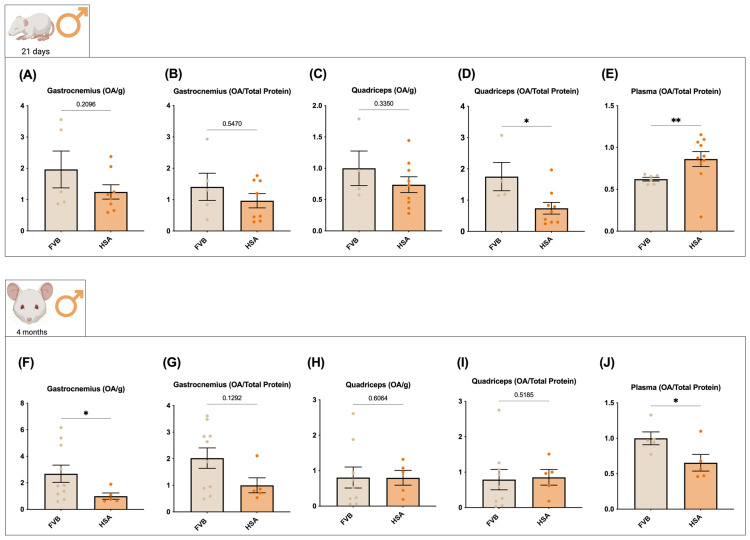
OA levels in male FVB and HSA^LR^ mice at postnatal day 21 and 4 months. Gastrocnemius and quadriceps muscles were dissected at both ages and OA was quantified by LC/MS. For each muscle, values are shown normalized to wet muscle weight (**A**,**C**,**F**,**H**) and to total protein content (**B**,**D**,**G**,**I**). Plasma OA was also quantified and normalized to total protein (**E**,**J**). Sample sizes: FVB (*n* = 6 at P21 and *n* = 10 at 4 months) and HSA^LR^ (*n* = 10 at P21 and *n* = 7 at 4 months). Bars show ± SEM with individual data points. Statistics: two-tailed unpaired Student’s *t*-test (equal variances), Welch’s *t*-test (unequal variances), or Mann–Whitney (non-normal data) as appropriate; significance: *p* < 0.05 (*) and *p* < 0.01 (**).

In males at P21, OA levels were lower in HSA^LR^ than in FVB muscle across readouts (Figure 2A–D), reaching significance in the quadriceps when normalized to total protein (FC = 0.42, corresponding to a 57.8% reduction; *p* < 0.05; Figure 2D). At this age, plasma OA was higher in HSA^LR^ than in FVB (FC = 1.39, corresponding to a 38.6% increase; *p* < 0.01; Figure 2E). By 4 months, OA was significantly reduced in the HSA^LR^ gastrocnemius when normalized to muscle weight (FC = 0.37, corresponding to a 62.8% reduction; *p* < 0.05; Figure 2F), with a nonsignificant downward trend when normalized to total protein (Figure 2G). The quadriceps showed no significant genotype differences by either normalization strategy (Figure 2H–I), whereas plasma OA was significantly reduced in HSA^LR^ at 4 months (FC = 0.65, corresponding to a 34.5% reduction; *p* < 0.05; Figure 2J).

**Figure 3:**
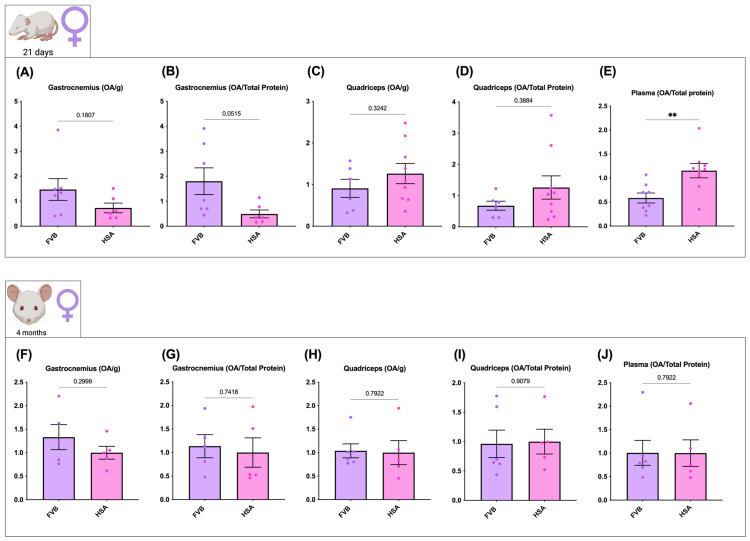
OA levels in female FVB and HSA^LR^ mice at postnatal day 21 and 4 months. Gastrocnemius and quadriceps muscles were dissected at both ages and OA was quantified by LC/MS. For each muscle, values are shown normalized to wet muscle weight (**A**,**C**,**F**,**H**) and to total protein content (**B**,**D**,**G**,**I**). Plasma OA was also quantified and normalized to total protein (**E**,**J**). Sample sizes: FVB (*n* = 8 at P21 and *n* = 6 at 4 months) and HSA^LR^ (*n* = 9 at P21 and *n* = 5 at 4 months). Bars show ± SEM with individual data points. Statistics: two-tailed unpaired Student’s *t*-test (equal variances), Welch’s *t*-test (unequal variances), or Mann–Whitney (non-normal data) as appropriate; *p* < 0.01 (**).

In females, effects were weaker and did not reach statistical significance in muscle. At P21, OA levels in the gastrocnemius and quadriceps showed no significant genotype differences under either normalization method (Figure 3A–D), although the gastrocnemius normalized to total protein approached significance (*p* = 0.0515; Figure 3B). As in males, plasma OA was higher in HSA^LR^ than in FVB (FC = 1.97, corresponding to a 97.3% increase; *p* < 0.01; Figure 3E). At 4 months, no significant genotype differences were observed in muscle or plasma (Figure 3F–J).

Overall, these data do not support a uniform, systemic OA deficit in HSA^LR^ mice. Instead, OA differences are modest and context-dependent, varying by sex, age, tissue and normalization method (muscle weight versus total protein), with the clearest reductions observed in males at specific time points and tissues. Nevertheless, subtle OA alterations cannot be ruled out in DM1, given the method-sensitive and tissue-specific signals observed.

### 2.3. Summary Profiles of OA Variation in HSA^LR^ vs. FVB

**Figure 4:**
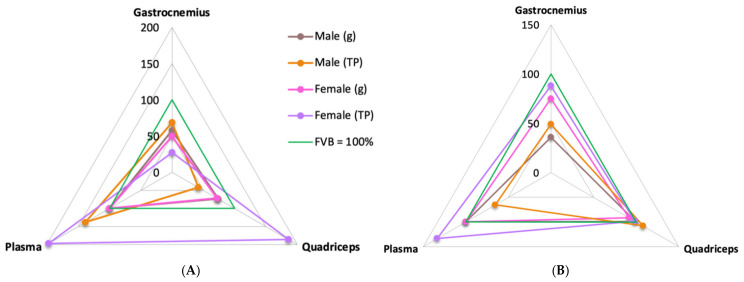
Radar plots of OA in HSA^LR^ relative to FVB. Sex comparison at two ages: (**A**) P21 and (**B**) 4 months. Vertices correspond to the gastrocnemius, quadriceps, and plasma. Lines show the HSA^LR^ mean expressed as a percentage of the corresponding FVB mean (FVB = 100%) calculated within the same sex, age, tissue and normalization. The 100% baseline is indicated by the green reference outline. Four series are plotted: male (g), male (TP), female (g), and female (TP). Plasma cannot be normalized to tissue weight; therefore, the plasma (g) vertex was fixed at 100% only to close the polygon and is not interpretable. Abbreviations: g, normalization to wet muscle weight; TP, normalization to total protein.

To summarize patterns across tissues and normalization strategies, we used radar plots displaying mean OA levels in HSA^LR^ as a percentage of the corresponding FVB mean (FVB = 100%) (Figure 4). This representation provides a relative effect-size overview, where values below 100% indicate reductions and values above 100% indicate increases relative to the matched FVB group. At P21 (Figure 4A), values were generally below 100% in muscle. In the gastrocnemius, all plotted series were <100%, with the largest decrease under total protein normalization and a smaller but consistent decrease when normalized to muscle weight (g). In the quadriceps, most series were at or below 100%, whereas the female total protein series showed a marked elevation (~200%). In plasma (normalized to total protein), OA was higher in HSA^LR^ than in FVB, with a larger increase in females (~200%) than in males (>100%). Plasma cannot be weight-normalized, so no plasma (g) measurement was taken. For plotting symmetry, the plasma (g) vertex was set to 100% solely to close the polygon and is not interpretable. By 4 months (Figure 4B), most values clustered closer to the baseline: the gastrocnemius remained below 100%, particularly in males under weight-based normalization, whereas the quadriceps and plasma (total protein) were near 100%, indicating that the P21 elevations were not sustained. Overall, the radar plots do not support a global OA deficit but instead highlight context-dependent differences, with the most consistent reductions observed in the gastrocnemius across ages.

## 3. Discussion

We studied alterations in lipid pathways and OA levels in DM1 to elucidate its potential as a candidate marker of DM1-like muscle pathology in HSA^LR^ mice. In our RNA-seq functional enrichment re-analysis, fatty-acid metabolic GO terms were consistently over-represented among downregulated genes in HSA^LR^ muscle across independent public datasets. Mapping DEGs onto an OA/glycerolipid pathway framework further localized the dominant transcript changes to nodes linked to MUFA/OA production and lipid handling. In HSA^LR^ mice, endogenous OA levels displayed selective reductions whose magnitude depended on muscle, sex, age, and the normalization strategy. To limit bias from disease-related atrophy and shifts in tissue composition, we therefore report OA both per muscle weight (tissue-level abundance) and per total protein (approximate cellular content), as the chosen normalizer can influence apparent effect sizes and, in some cases, interpretation [33,34]. Wet tissue weight normalization reflects OA abundance relative to the whole tissue specimen, but may be affected by non-contractile components such as extracellular matrix expansion, fat infiltration, or changes in water content. Total protein normalization may better approximate OA abundance relative to the cellular/protein-rich fraction of the sample; however, this denominator may also be influenced by disease-associated remodelling, including fibre-type switching, changes in contractile protein abundance, proteostasis alterations, and overall tissue cellularity. Thus, protein-normalized OA should be interpreted as a cellular-content-adjusted estimate rather than a direct measurement of intracellular OA concentration. Taken together, the enrichment patterns, pathway context, and context-dependent OA changes found are consistent with disturbed MUFA/OA homeostasis, but do not support a uniform OA deficit; rather, they point to several non-mutually exclusive mechanisms that will require direct functional testing.

At P21, plasma OA was higher in HSA^LR^ than in FVB in both sexes, and this difference was no longer evident at 4 months, consistent with the weaning transition, when dietary input still contributes substantially to circulating OA levels. Mammalian milk is oleate-rich (~30% FA in humans), creating a context in which circulating OA can be transiently higher and small genotype differences in uptake, transport and/or partitioning may become more detectable under high exogenous OA flux [35,36]. Across weaning, mice undergo a developmental shift in Δ9-desaturation control, with Scd2 prominent in embryos/neonates and greater reliance on Scd1 in adult metabolic tissues. Accordingly, the balance between dietary OA and endogenous 18:0→18:1 conversion can vary by sex and muscle without requiring changes in *Scd1* mRNA [37,38]. In our re-analysis of three independent post-weaning male HSA^LR^ RNA-seq datasets comparing HSA^LR^ with age-matched FVB (10–12 weeks and <4 months), the OA/glycerolipid pathway map consistently highlighted *Scd2*, whereas *Scd1* remained comparatively unchanged. This differs from previous in vitro observations in human DM1 muscle cells where reduced endogenous OA was linked to decreased SCD1 levels [14]. We interpret this discrepancy as potentially reflecting species- and model-specific regulation rather than a direct contradiction. In mice, Scd1 and Scd2 display tissue- and developmental-context dependence, with Scd2 playing important roles during early development and lipid synthesis [37,38]. In addition, our data derive from whole skeletal muscle in vivo, where fibre composition, muscle-specific transgene load, tissue remodelling and systemic metabolic state may influence lipid-handling gene expression, unlike cell-autonomous in vitro systems. Thus, the consistent Scd2 signal in HSA^LR^ muscle suggests isoform-selective remodelling of the Δ9-desaturation module and raises the possibility that Scd2 contributes more prominently to murine skeletal muscle OA homeostasis under disease conditions than previously appreciated. However, transcript abundance does not necessarily reflect enzymatic flux, since Δ9-desaturation also depends on cytochrome b5/b5-reductase activity, substrate availability and cellular redox state [16,39]. Therefore, this observation should be considered hypothesis-generating, and Δ9-desaturation indices such as 18:1/18:0, ideally resolved by lipid class, will be needed to distinguish reduced desaturation from increased OA utilization, oxidation, storage or rerouting into membrane lipids [40].

By 4 months, dietary effects fade and OA pools likely reflect flux (desaturation, β-oxidation/lipophagy and partitioning into membranes vs. storage) modulated by sex-specific muscle physiology (e.g., enzyme programmes and substrate use) [41]. Importantly, omics profiling at nearby ages shows higher *HSA^LR^* transgene expression and more pronounced MBNL-dependent mis-splicing in the gastrocnemius than in the quadriceps [29]. Such muscle-specific transgene load and processing defects can subtly remodel fibre-type balance and oxidative capacity [42,43], autophagy [44,45], and extracellular matrix (ECM) content [46], thereby changing both lipid partitioning and the denominator used for quantification (per gram of tissue vs. per total protein). This provides a direct rationale for why statistical significance can flip between normalizations. Consequently, an OA shortfall may be evident under one metric but not the other, consistent with the normalization-sensitive OA signals across muscles in HSA^LR^ and the stronger, more reproducible trends in the gastrocnemius compared with the quadriceps.

Other nodes within the OA/glycerolipid schematic also point to altered lipid handling at steps upstream of bulk OA accumulation, but these signals were less consistent than the desaturation node. In particular, transcripts annotated to fatty-acid uptake/activation (including *Slc27a2*/*FATP2*) were decreased in a contrast-dependent manner in the re-analyzed HSA^LR^ muscle datasets, which is compatible with remodelling of fatty-acid handling capacity in vivo [32]. Although FATP2 is most prominently expressed in the liver and kidney and is not generally considered a dominant fatty-acid uptake route in skeletal muscle, it remains mechanistically notable because SLC27/FATP proteins can couple long-chain fatty-acid transport with acyl-CoA synthetase activity, thereby linking uptake to intracellular activation and metabolic trapping [32]. Thus, reduced Fatp2 expression could reflect altered fatty-acid handling capacity in HSA^LR^ muscle, but should not be interpreted as direct evidence that OA import is globally reduced. Skeletal muscle expresses multiple, partially redundant fatty-acid uptake and handling systems, including FAT/CD36, FABPpm and other FATP isoforms, which can be coordinately regulated by insulin and muscle contraction [47]. Therefore, given the non-uniform Fatp2 signal across datasets, we interpret Fatp2 downregulation as a context-dependent component of broader lipid remodelling rather than a dominant driver of the OA phenotype. Based on the current transcriptomic data, we cannot determine whether altered uptake/activation contributes more to the OA changes than Δ9-desaturation or downstream OA utilization. Glycerolipid synthesis transcripts, including GPAT/AGPAT/DAG nodes, showed only small and dataset-dependent changes, consistent with isoform redundancy. Therefore, functional assays of fatty-acid uptake, acyl-CoA formation and lipid-class-resolved lipidomics, for example distinguishing triacylglycerols from phospholipids, will be required to resolve whether context-dependent OA signals reflect altered uptake, storage, membrane composition or turnover.

The context-dependent OA changes observed here also refine the mechanistic model proposed from previous DM1 cell studies linking OA availability to the MSI2/miR-7/autophagy axis. In those systems, reduced OA was associated with impaired miR-7 processing, MSI2-dependent repression of miR-7, autophagy dysregulation and defective myogenesis. In contrast, the present HSA^LR^ in vivo data do not show a uniform OA deficit, and prior work indicates that HSA^LR^ muscle does not exhibit overt MSI2 overexpression or robust activation of the MSI2/miR-7/autophagy programme at baseline, whereas forced MSI2 expression can induce autophagy, fibre atrophy and weakness [48]. Together with the comparatively mild atrophy of HSA^LR^ muscle [49], this suggests that the baseline HSA^LR^ model may only partially recapitulate the OA-MSI2/miR-7/autophagy phenotype observed in human DM1 cellular systems. Therefore, the apparent discrepancy may reflect differences between human cell-autonomous DM1 models and whole murine skeletal muscle in vivo, as well as differences in disease severity, developmental stage, metabolic context or transgene-driven pathology. Since MSI2, miR-7 and autophagy markers were not directly measured in the same samples, the activation of this axis in vivo remains a testable hypothesis rather than a conclusion of the present study. Future studies should directly assess whether OA depletion or supplementation modulates MSI2, miR-7 and autophagy readouts in HSA^LR^ muscle and in complementary DM1 models.

Sex differences may contribute to the stratified patterns we observed, but our data do not support a uniform, muscle-intrinsic sex effect. In males, OA reductions were clearer and more consistent across specific tissues and normalization strategies, and this directionality is supported by two independent layers of evidence: our in vivo OA quantification at P21 and 4 months and the male-only public RNA-seq re-analyses, which localize transcript-level changes to fatty-acid metabolic processes and the Δ9-desaturation module. These male-weighted signals are compatible with sex-dependent severity reported in DM1 cohorts [50] and with well-established sexual dimorphism in muscle enzyme activities and transcriptional programmes that can bias lipid handling under disease pressure [51]. However, this interpretation should be tempered by reports that female HSA^LR^ mice can exhibit broadly comparable muscle functional, histological, and molecular phenotypes to males, with differences reported mainly in plasma biochemistry [52]. Together, these observations suggest that any sex-related effects on OA are likely modest and contingent on muscle, age, systemic milieu, and normalization strategy, rather than robust across endpoints. Accordingly, sex-stratified designs remain important to quantify heterogeneity, measure transgene load by sex and muscle, and pair these measurements with lipidomics in the same animals to distinguish biological variability from sampling or normalization artefacts [50,53].

Finally, human studies show that Δ9-desaturation indices (18:1/18:0; 16:1/16:0), commonly used in vivo proxies of net Δ9-desaturation/handling, are sensitive to dietary fat quality and are associated with lifestyle factors and body composition/adiposity [54,55]. In parallel, ageing is accompanied by broad metabolic remodelling, including changes in skeletal muscle mitochondrial capacity and substrate utilization that can influence MUFA pools through altered oxidation and turnover, independently of Δ9-desaturase mRNA [56,57]. OA-specific data support this concept: oleate oxidation in primary human myotubes varies with donor age [58], skeletal muscle phospholipid OA decreases across childhood [59], and in adults, skeletal muscle fatty-acid composition (including OA) reflects dietary fat composition [60]. Together, these observations indicate that age, diet, and metabolic status shape both production and utilization of OA, supporting the idea that the context-dependent OA signals observed in HSA^LR^ muscle could reflect altered flux through the desaturation/handling network (including isoform-selective regulation such as *Scd2*) even when *Scd1* transcripts remain stable.

Clinically, dyslipidemia and insulin resistance are common in DM1, so an OA shortfall could in theory contribute to membrane and ER stress, yet this has not been tested in patients, and no controlled trials of MUFA-rich diets or OA supplementation in DM1 have been reported [9,53]. The present in vivo data do not rule out the therapeutic potential of OA supplementation, but rather indicate that OA homeostasis in HSA^LR^ mice is context-dependent and does not reflect a uniform systemic deficit. Therefore, OA supplementation remains worth evaluating in controlled preclinical studies that account for dietary background, age, sex and muscle-specific responses. Nonetheless, MUFAs can improve insulin signalling and stress responses in other settings, supporting careful preclinical testing in DM1 models before translation [10,12].

In conclusion, HSA^LR^ mice exhibit selective, context-dependent reductions in OA in skeletal muscle that vary by muscle, sex, age, and normalization, with the clearest signals found in males. Given the context-dependent nature of these changes, OA should be regarded as a candidate marker rather than a validated biomarker of DM1-like muscle pathology. Although RNA-seq pathway mapping implicates the Δ9-desaturation module (notably *Scd2*), the pattern overall points to flux-level control, via substrate/cofactor availability, redox state, transport, and utilization, rather than a simple transcriptional block of the canonical Scd1 isoform [16,24]. Thus, the data do not support a global OA deficit but do identify perturbed OA homeostasis as a model-sensitive indicator of DM1-like muscle biology.

## 4. Materials and Methods

### 4.1. Animals and Experimental Design

HSA^LR^ mice (homozygous for the HSA^LR^ transgene) carry the human skeletal alpha-actin (*HSA*) transgene with an expanded CTG region in the 3′UTR, producing toxic CUG repeat RNA specifically in skeletal muscle. HSA^LR^ and FVB wild-type controls were bred and housed in our animal facility, with *ad libitum* access to water and standard chow (Teklad Global 14% Protein Maintenance Diet, 2014; Inotiv/Envigo, Indianapolis, IN, USA). All animals were sampled under comparable nutritional conditions. Mice had *ad libitum* access to food and water until sacrifice. Sample collection was performed during the same time window to minimize circadian and feeding-related variability.

Animals were grouped by sex and age: at postnatal day 21 (P21) and 4 months (P120). P21 was included to determine whether OA alterations could be detected early, before the appearance of a fully established adult muscle phenotype. The 4-month time point was selected because it falls within the adult window in which disease-associated molecular, histological and functional alterations have been reported in HSA^LR^ mice, while still preceding the overt muscle weakness described at later adult ages [25,52,61]. At 4 months, the groups included *n* = 7 HSA^LR^ and *n* = 10 FVB males, and *n* = 5 HSA^LR^ and *n* = 6 FVB females. At P21, there were *n* = 10 HSA^LR^ and *n* = 6 FVB males, and *n* = 9 HSA^LR^ and *n* = 8 FVB females. HSA^LR^ transgenic mice, originally described by [25], were generously provided by Dr. Charles Thornton (University of Rochester, NY, USA). Sample size was determined using G Power v3.1 software.

### 4.2. Sample Collection and Lipid Extraction

Mouse blood obtained via cardiac puncture was collected in heparinized tubes and centrifuged at 3000× *g* for 10 min at 4 °C. The plasma supernatant was isolated and stored at −80 °C. For lipid extraction, 30 μL of plasma was mixed with cold methanol (#15518534, Thermo Fisher Scientific, Waltham, MA, USA) and methyl-tert-butyl ether (MTBE; #143312, Panreac Applichem ITW Reagents, Barcelona, Spain). After biphasic separation with water, the upper organic phase was collected and dried using a SpeedVac.

Gastrocnemius and quadriceps muscles were excised, snap-frozen in liquid nitrogen, and stored at −80 °C. Approximately 80 mg of frozen tissue was homogenized in 1 mL of cold methanol using a tissue homogenizer on ice. Next, 500 μL of cold methanol and 5 mL of cold MTBE were added, followed by vortexing and incubation for 1 h at 4 °C under agitation. Phase separation was induced by adding 1.25 mL of ddH_2_O, vortexing for 20 s, and incubating at room temperature for 10 min. After centrifugation (1100× *g*, 10 min), the upper organic layer (apolar lipids) was collected and dried. The remaining aqueous phase was re-extracted with 2 mL of MTBE and a MeOH:H_2_O (10:3:2.5) mixture. The new upper phase was also collected and dried. The final aqueous fraction was precipitated with 4 volumes of cold methanol, incubated at −20 °C for 1 h, and centrifuged at 13,000× *g* for 12 min at 4 °C. The resulting pellet (polar proteins) was resuspended in RIPA buffer (#10230544, Thermo Fisher Scientific) supplemented with protease (#116974898001, Roche Applied Science, Indianapolis, IN, USA) and phosphatase inhibitor cocktails (#4906837001, Roche Applied Science).

### 4.3. Oleic Acid Quantification

OA concentration was determined using a liquid chromatography–mass spectrometry (LC/MS) system (ACQUITY TQD, Waters, MA, USA). Chromatographic separation was performed using an ACQUITYUPLC C18 Kinetex column (Phenomenex, particle size 1.7 μm; 2.1 mm × 100 mm). The mobile phase was in isocratic mode and consisted of MeOH: CHCl3: H2O (1:1:0.04). The flow rate used was 0.2 mL/min. The mass spectrometer was equipped with a Z-spray electrospray ionization source, and samples were analyzed under the following conditions: capillary, 3 KV; cone, 40 V; extractor, 5 V; RF Lens, 0.3 V; source temperature, 120 °C; desolvation temperature, 300 °C; cone gas, 25 L/h; desolvation gas flow, 650 L/h. MS1 parameters were: LM resolution, 13; HM resolution, 13; ion energy, 1. MS2 parameters were: LM resolution, 13; HM resolution, 13; ion energy, 1; multiplier, 650 V. Spectra were acquired in negative-ionization selected reaction monitoring (SRM) mode with an inter-channel delay of 0.050 s. OA was quantified using an external calibration curve prepared with an oleic acid analytical standard (#O-1008, Sigma-Aldrich, St. Louis, MO, USA). The calibration curve covered a concentration range of 0.00003–0.015 mM, and sample concentrations were calculated within the range of this curve. Therefore, the LC-MS output corresponded to absolute OA concentrations calculated against an external standard curve, rather than relative signal intensities. No stable isotope-labelled OA or other internal standard was used for quantification. Consequently, OA values were not corrected for extraction recovery or extraction efficiency. According to the LC-MS facility workflow, samples were injected as received, and no additional dilution factor was applied during LC-MS analysis.

For muscle tissues, OA levels were reported per wet tissue weight and per total protein. Total protein was quantified by bicinchoninic acid (BCA) assay following the manufacturer’s protocol (#10741395, Thermo Fisher Scientific). Dual normalization was pre-specified to mitigate bias from disease-related atrophy and compositional remodelling. Since the choice of biomass normalizer can alter apparent effect sizes, both normalization approaches are presented and interpreted accordingly [34]. Importantly, normalization to wet tissue weight or total protein content was used to account for differences in biological sample input, but does not correct for extraction efficiency.

### 4.4. RNA Sequencing Analysis

Raw RNA-seq data from [27,28,29] were downloaded from their respective public repositories. Libraries were trimmed using Fastp v.1.0.1 [62] and aligned using STAR v2.7.11b [63] against the GRCm39 genome assembly. The resulting BAM files were used to generate raw count matrices using FeatureCounts v2.1.1 [64]. Finally, data normalization and differential-expression analyses were conducted using the DESeq2 R package [65]. Genes with an adjusted *p*-value of less than 0.05 and absolute log_2_FC value greater than log_2_(1.5) were selected as differentially expressed. Enrichment analyses were conducted using ClusterProfiler R package [66], selecting as significant those terms with a Benjamin–Hochberg FDR < 0.05.

### 4.5. Statistical Analysis

Normality was assessed with the Shapiro–Wilk test, and homogeneity of variances with an F test. Outliers were identified and removed using the ROUT method (Q = 1%). A total of 13 individual outlier values were excluded from the OA analyses; these corresponded to specific tissue- and normalization-based measurements rather than complete animal exclusion. The number of excluded values and their corresponding experimental groups are reported in Supplementary Table S1. For comparisons between two independent groups, a two-tailed unpaired Student’s *t*-test was used when data were normally distributed and variances were equal; when variances differed, the Welch *t*-test was applied; when normality was not met, the Mann–Whitney U test was used. Statistical significance was set at *p* < 0.05. Results are reported as mean ± SEM for parametric analyses and as median for nonparametric analyses.

For OA quantification, effect sizes were calculated as fold change relative to the corresponding age-, sex- and tissue-matched FVB group (FC = mean HSA^LR^/mean FVB) and as percentage change relative to FVB. For RNA-seq pathway genes, effect sizes are reported as log_2_FC values from the differential-expression analyses.

## Data Availability

The original contributions presented in this study are included in the article. Further inquiries can be directed to the corresponding author. RNA-seq libraries were downloaded from public repositories. SRA accessions SRR7707863, SRR7707864, SRR7707865, SRR9720668, SRR9720669, SRR9720670, SRR9720665, SRR9720666, SRR9720667, SRR9720663, SRR9720664, SRR9720672, SRR9722308, SRR9722309, SRR9722310, SRR9720661, SRR9720662, and SRR9720671 were downloaded from [28]. SRA accessions SRR31749926, SRR31749927, SRR31749928, SRR31749929, SRR31749930, SRR31749931, SRR31749932, SRR31749933, SRR31749934, SRR31749935, SRR31749936 and SRR31749937 were downloaded from [29]. Finally, ref. [30] data was downloaded from the E-MTAB-10842 Accession from the European Nucleotide Archive.

## Supplementary Materials

The following supporting information can be downloaded at: https://www.mdpi.com/article/10.3390/ijms27104211/s1.
