# Supplementary material for: Oleic Acid Levels in HSALR Mouse Model of Myotonic Dystrophy Type 1
Source: Int J Mol Sci. 2026 May 9;27(10):4211. doi: 10.3390/ijms27104211 (PMC13206852; doi:10.3390/ijms27104211)
Supplement: Supplementary file 1 [file ijms-27-04211-s001.zip › ijms-4281662-supplementary.pdf]

Supplementary Materials

Table S1. Summary table of outliers detected by ROUT. Outliers were identified using the ROUT method with Q = 1%. Only groups in which outlier values were detected are shown. Outlier values correspond to individual OA measurements within each tissue- and normalization-specific analysis and do not necessarily imply complete exclusion of the corresponding animal from all analyses. Groups not listed had no outlier values excluded.

**Sex**	**Age**	**Tissue**	**Normalization**	**Genotype/group**	**Initial *n***	**Outlier values excluded**	**Final *n***
Female	P21	Gastrocnemius	OA/g tissue	HSA^{LR}	9	1	8
Female	P21	Quadriceps	OA/ total protein	FVB	8	3	5
Female	4 months	Gastrocnemius	OA/g tissue	FVB	6	1	5
Male	4 months	Gastrocnemius	OA/g tissue	HSA^{LR}	7	2	5
Male	4 months	Gastrocnemius	OA/ total protein	HSA^{LR}	7	2	5
Male	4 months	Quadriceps	OA/ total protein	FVB	10	1	9
Male	4 months	Quadriceps	OA/g tissue	FVB	10	1	9
Male	4 months	Quadriceps	OA/g tissue	HSA^{LR}	7	1	6
Male	4 months	Quadriceps	OA/total protein	HSA^{LR}	7	1	6
